# Prevalence and associated factors of antenatal depression in rural Bangladesh

**DOI:** 10.1371/journal.pone.0321965

**Published:** 2025-04-23

**Authors:** Rifa Tamanna Mumu, Dipak Kumar Mitra, Md Parvez Shaikh

**Affiliations:** 1 Department of Public Health, School of Health and Life Science, North South University, Bashundhara, Dhaka, Bangladesh; 2 Department of Industrial & Production Engineering, Shahjalal University of Science and Technology, Sylhet, Bangladesh; Kandahar University, Faculty of Medicine, AFGHANISTAN

## Abstract

**Background:**

According to the World Health Organization (WHO), approximately 322 million individuals all over the world suffered from depressive disorders in 2015. The risk of depression increases in pregnancy due to certain hormonal changes in the body. Despite the severe impacts of antenatal depression on both maternal and infant health, research on this issue remains limited in Bangladesh.

**Objective:**

To identify the prevalence and associated factors of antenatal depression in rural Bangladesh.

**Method:**

A cross-sectional study was conducted from January 08 to January 14, 2024, in Lohagara, a rural subdistrict of Narail in southern Bangladesh. The study recruited 350 pregnant women in different trimesters who attended antenatal checkups at a government health complex and a private hospital. Data was collected by face-to-face interviews using the Bengali-translated version of the Edinburgh Postnatal Depression Scale (EPDS) and another structured questionnaire. Pearson’s chi-square test, bivariate, and multivariate logistic regression were conducted to identify associated factors. Data were analyzed using STATA version 14.

**Result:**

The point prevalence of antenatal depression was 39% (38.9%, 95% CI = 33.9% to 44%). Gestational week (AOR = 0.4, 95% CI = 0.2, 0.8), unintended pregnancy (AOR = 1.7, 95% CI = 1, 3), intimate partner violence (AOR = 3.3, 95% CI = 1.1, 9.7), a history of previous diseases (AOR = 2.4, 95% CI = 1.1, 5.2), and having polygamous husbands (AOR = 13.6, 95% CI = 1.1, 164) were found significantly associated with the development of depression in pregnancy.

**Conclusion:**

The high prevalence of prenatal depression in rural Bangladesh highlights the urgent need for effective intervention. Raising awareness among healthcare professionals and families of pregnant women is essential to reducing its impact. Strategic planning and policymaking are necessary to address underlying social issues such as polygamy and intimate partner violence. Additionally, providing enhanced counseling and care for women with unplanned pregnancies or pre-existing health conditions is crucial for improving maternal mental well-being.

## Introduction

Depression is a widespread mental health disorder characterized by persistent low mood, concentration difficulties, low self-esteem, changes in appetite and sleep patterns, loss of interest in activities, significant weight fluctuations, feelings of hopelessness, and recurrent thoughts of death. It is one of the top five contributors to the global disease burden [1]. Projections indicate that by 2030, depressive disorders will be among the three leading causes of the global health burden [2].

In 2015, the World Health Organization (WHO) reported that 322 million people were affected by depressive disorders globally, with 27% from the Southeast Asian region [1]. The risk of mental disorders, especially depression, is higher in women than in men [3]. During pregnancy, this risk further increases due to certain hormonal changes in the body [4]. The global prevalence of antenatal depression varies widely, ranging from 15% to 65% [5]. In high-income countries, the prevalence ranges between 5% to 30% [6–8], while in low-income countries, it ranges from 15.6% to 31.1% [9–11]. Among South Asian women, 17.5% suffer from antenatal depression [12]. A recent study found a 78.5% prevalence among Afghan pregnant women aged 15–45 years, with associated factors including 30–45 years of age, unemployment, low socioeconomic condition, no traumatic incidence in the previous month, and last week’s sex deprivation [13].

The prevalence of prenatal depression in Bangladesh ranges from 18% to 33% [14,15], making it a significant public health concern. A recent study conducted in a rural sub-district in Matlab, Bangladesh, reported a 33% prevalence of depression among women in their 34^th^ to 35^th^ week of gestation. Associated factors included lack of support from husband or mother-in-law, domestic violence, and family pressure to give birth to a male child [14]. Similarly, a cross-sectional study in rural Sylhet identified husbands’ preferences for male babies, low family support, and sexual violence as major contributors to antenatal depression [16].

Prenatal depression can have severe consequences, affecting both mothers and newborns. Depressed pregnant women may produce elevated levels of cortisol, a hormone that can negatively impact fetal growth and brain development [17]. Additionally, women experiencing prenatal depression face a higher risk of developing hyperemesis gravidarum, which increases the likelihood of a miscarriage, low birth weight, and preterm birth [18]. They are also more prone to substance abuse, preeclampsia, hemorrhage, edema, postpartum depression, and severe headaches [19, 20]. Infants born to mothers with prenatal depression are at a greater risk of low birth weight (LBW), low mean APGAR scores at 1 and 5 minutes after birth, and even premature mortality [21,22].

Antenatal depression presents a significant challenge for expectant mothers and their babies, particularly in Bangladesh. While some studies examined prenatal depression in urban areas, research on rural populations remains limited. Furthermore, there is a lack of comprehensive data on depression and its effects across different trimesters in pregnancy in Bangladesh. In this study, we explored the prevalence of antenatal depression and factors associated with its development in rural Bangladesh. Findings from this study can play a pivotal role in formulating specific policies and interventions and organizing health promotional and educational programs aiming at raising awareness among rural communities, stakeholders, and policymakers to prevent prenatal depression.

## Materials and methods

### Study design and setting

A cross-sectional study was conducted from January 08 to January 14, 2024, in a government hospital named Upazila Health Complex, Lohagara, and a private hospital named Khan General Hospital, Lahuria. Both hospitals were located in Lohagara, a rural sub-district of Narail in the southern part of Bangladesh.

### Study participants

The target population was pregnant women in any trimester in Lohagara, and the sample population was pregnant women in different trimesters attending ANC corners of both hospitals for antenatal checkups during the study period.

### Sample size and sampling technique

Considering a 33% prevalence of antenatal depression in Bangladesh [14] at a 95% confidence interval (CI) with a 5% margin of error, the calculated sample size is: $n={\left(1.96\right)}^{2}\times \left[\frac{0.33*\left(1-0.33\right)}{{\left(0.05\right)}^{2}}\right]=340.$

A total of 350 participants were recruited by systematic sampling to minimize bias. Every third patient attending antenatal checkups in both hospitals was selected as a participant for the interview.

### Data collection and measurement tools

The presence of depression was assessed by the Bengali-translated version of the Edinburgh Postnatal Depression Scale (EPDS-B) [23–25]. The questionnaire consists of ten questions with a total score ranging from 0 to 30. A score of 10 or higher indicated probable antenatal depression [12,26,27].

Another structured questionnaire, translated into Bengali, was used to collect participants’ sociodemographic, obstetric, psychosocial, psychological, disease, and treatment-related data. These questionnaires were pretested on the target population rather than the study participants, and necessary adjustments were made before data collection.

We collected data through face-to-face interviews with each participant. Those who met the eligibility criteria were involved in a 15-minute interview providing informed written consent. We selected one separate room in each hospital for interviews where only the interviewer and interviewee were present. After collecting data daily, we cross-checked them, identified possible errors, and corrected them.

### Data management & analysis

Data were analyzed using STATA version 14. Pearson’s chi-square test was performed to identify possible associations between variables. A binary logistic regression was also applied to find crude odds ratios. Variables with p-values <0.05 were considered for multivariate analysis by multinomial logistic regression to adjust confounding factors. Statistical significance was determined by p-values<0.05, and the strength of association was assessed by adjusted and unadjusted odds ratios, and their 95% CIs.

### Ethical considerations

Ethical approval was obtained from the Institutional Ethics Committee of North South University before data collection (Approval Number: 2023/OR-NSU/IRB/1224). Permission letters were also received from Upazila Health Complex, Lohagara, and Khan General Hospital, Lahuria. Informed written consent was taken from pregnant mothers aged 18 years or older. In cases of participants under 18, their legal guardians provided informed written consent before the interview. Respondents were assured of the confidentiality of the information and informed about the study’s purpose, advantages, and potential risks.

## Result

### Socio-demographic characteristics

Data were collected from 350 pregnant women, with a response rate of 98.2%. Participants had a median age of 23, with most of them (83.7%) aged between 18 and 30. About 96.3% were Muslim by religion, and 7.4%(26) never attended school. All of the respondents were married, and 4% were employed. More than half of the women (58%) had a monthly family income ranging from 10,000–20,000 BDT (83–167 USD).

### Obstetric characteristics

Approximately, 53.4% of the women were in their second trimester of pregnancy. The median age of marriage was 18 years, with an interquartile range of 16–19 years. Additionally, 38.6% (135) of the expectant mothers were pregnant for the first time.

Among the multiparous women, 51% (105) underwent at least one cesarean section, and 30.6% (63) experienced complications during previous deliveries. Furthermore, 21.5% (75) reported a history of abortion, stillbirth, or intrauterine fetal death.

### Psychosocial characteristics

Among the participants, 74% (259) planned their pregnancies, while 26% (91) did not. A small portion (4.8%) reported poor relationships with their husbands, while 6% () had strained relationships with their in-laws. In 1.4% of cases (), either their parents-in-law passed away or were not living with them.

Approximately 5.1% (19) of participants experienced domestic violence, and 5.4% () were victims of sexual violence. Around 10% () reported experiencing intimate partner violence during their current pregnancy.

Regarding gender-related expectations, 18.6% (65) of participants indicated that their husbands preferred having male children and 18% (63) were under pressure from in-laws to give birth to a male child.

### Disease and treatment-related history

Among the participants, 10.6% () reported having pre-existing medical conditions such as diabetes mellitus, hypertension, bronchial asthma, or thyroid disorders before pregnancy. Additionally, 14% (49) had a history of at least one surgery other than the cesarean section.

### Psychological characteristics

Among the pregnant women, 2.6% () reported that their husbands had multiple wives, while 0.6% stated that their husbands were involved in extramarital relationships.

### Prevalence of depression among pregnant women

After calculating the total EPDS score, it was found that about 38.9% (95% CI = 33.9% to 44%) of women experienced depression during pregnancy **Table 1**.

**Table 1:** Prevalence of antenatal depression among pregnant women attending antenatal checkups (n=350).

Prevalence of Antenatal Depression			
**Depression**	**EPDS score**	**Number (n)**	**Percentage (%)**
Yes	10-30	136	38.9
No	0-9	214	61.1

**Fig 1:**
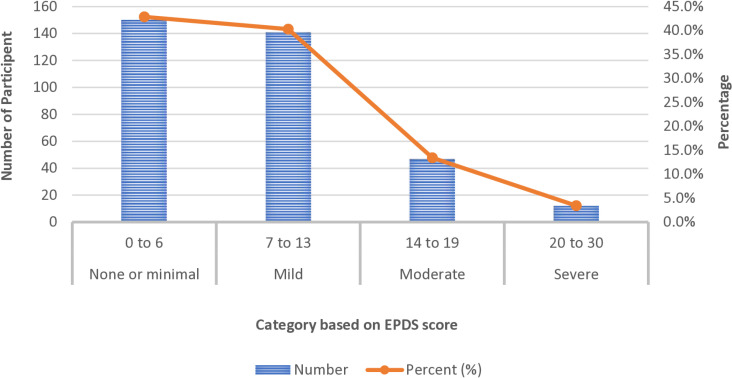
Extent of depression among pregnant women.

**Fig 1** presents that about 42.9% (150) of women had minimal or no depression (EPDS score 0–7), and 40.3% (141) had mild depression (EPDS score 7–13). Additionally, 12.2% (43) had an EPDS score between 14 and 19, indicating moderate depression, while 4.6% () scored over 19 signifying severe depression [28].

### Factors associated with antenatal depression

**Table 2** depicts that among the variables, gestational week (p= 0.026), the number of pregnancies (p=0.005), type of pregnancies (p= 0.002), relationship with husband (p= 0.017), and in-laws (p= 0.001), history of domestic (p=0.047) and sexual violence (p= 0.007), having multiple-married husbands (p= 0.002), and history of previous diseases (p= 0.007) were found statistically significant with a p-value <0.05 in the chi-square test.

**Table 2:** Associated factors of antenatal depression (n=350).

Variables	Category	No Depression	Mild Depression	Moderate Depression	Severe Depression	Chi2 p-value
Gestational week	<13 weeks	14 9.3%	30 21.3%	10 21.3%	2 16.7%	0.026
	13-28 weeks	92 61.3%	74 52.5%	15 31.9%	6 50%	
	29-40 weeks	44 29.3%	37 26.2%	22 46.8%	4 33.3%	
Para	Nulliparous	68 45.3%	48 34.4%	16 34.4%	3 25%	0.005
	Multiparous	82 54.7%	93 66%	31 66%	9 75%	
Type of pregnancy	Planned	123 82%	97 68.8%	35 72.3%	4 33.3%	0.002
	Unplanned	27 18%	44 21.2%	12 27.7%	8 66.7%	
Relationship with husband	Good	72 48%	60 42.6%	22 46.8%	3 25%	0.017
	Moderate	73 48.7%	78 55.3%	20 42.6%	5 41.7%	
	Poor	5 3.3%	3 2.1%	5 10.6%	4 33.3%	
Relationship with in-laws	Good	128 85.3%	110 78%	28 79.6%	3 25%	0.001
	Moderate	16 10.7%	24 17%	12 25.5%	3 25%	
	Poor	5 3.3%	4 2.8%	7 14.9%	5 41.7%	
	Dead/Separated	1 0.7%	3 2%	0	1 8.3%	
Domestic violence	Yes	5 3.3%	7 5%	4 8.5%	2 16.7%	0.047
	No	145 96.7%	134 95%	43 91.5%	10 83.3%	
Sexual violence	Yes	4 2.7%	9 6.4%	3 6.4%	3 25%	0.007
	No	146 97.3%	132 93.6%	44 93.6%	9 75%	
Polygamous husband	Yes	1 0.7%	4 2.8%	1 2%	3 25%	0.002
	No	149 99.3%	137 97.2%	46 97.9%	9 75%	
Previous disease	Yes	9 6%	18 12.8%	7 14.9%	3 25%	0.007
	No	141 94%	123 87.2%	40 85%	9 75%	

**Table 3** presents that after multivariate analysis, 60% less likelihood of depression was observed in the second trimester (AOR = 0.4, 95% CI = 0.2, 0.8) compared to the first. Mothers who experienced intimate partner violence were more likely (AOR = 3.3, 95% CI = 1.1, 9.7) to develop antenatal depression than those who didn’t. The adjusted odds ratio was about two times higher in women having unwanted pregnancies (AOR = 1.8, 95% CI = 1, 3) and 2.4 times higher in those with a history of previous diseases (AOR = 2.4, 95% CI = 1.1, 5.2). Additionally, the odds of antenatal depression were more than thirteen times greater in women having multiple married husbands (AOR = 13.6, 95% CI = 1.1, 164).

**Table 3:** Bivariate and multivariate associations of prenatal depression.

Variables	Category	Depression	No Depression	COR (95% CI)	AOR (95% CI)	p-value
Gestational week	<13 weeks	30 (53.6%)	26 (46.4%)	1	1	
	13-28 weeks	63 (33.7%)	124 (66.3%)	0.4 (0.2, 0.8)	0.4 (0.2,0.8)	**0.008**
	29-40 weeks	43 (40.2%)	64 (59.8%)	0.6 (0.3, 1.1)	0.6 (0.3, 1.2)	0.154
Para	Nulliparous	40 (29.6%)	95 (70.4%)	0.5 (0.3, 0.8)	0.9 (0.5,1.5)	0.575
	Multiparous	96 (44.7%)	119 (55.3%)	1	1	
Type of pregnancy	Planned	88 (34%)	171 (66)	1	1	
	Unplanned	48 (52.7%)	43 (47.3%)	2.2 (1.4, 3.6)	1.8 (1, 3.1)	0.057
Relationship with husband	Good	55 (35%)	102 (65%)	1	1	
	Moderate	69 (39.2%)	107 (60.8%)	1.2 (0.8, 1.9)	1.2(0.7, 1.9)	0.567
	Poor	12 (70.6%)	5 (29.4%)	4.5 (1.3, 13.3)	1.6 (0.3, 8.9)	0.592
Relationship with in-laws	Good	91 (33.8%)	178 (66.2%)	0.3 (0.06, 2.08)	2.6 (0.2, 38.7)	0.479
	Moderate	27 (49%)	28 (51%)	0.6 (0.1, 4)	4.7 (0.3, 71.7)	0.266
	Poor	15 (71.4%)	6 (28.6%)	1.7 (0.2, 12.6)	11.2 (0.6, 194)	0.1
	Dead/ Separated	3 (60%)	2 (40%)	1	1	
Domestic violence	Yes	11 (61.1%)	7 (38.9%)	2.6 (1, 6.9)	1.2 (0.3, 5.7)	0.804
	No	125 (37.7%)	207 (62.3%)	1	1	
Sexual violence	Yes	13 (68.4%)	6 (31.6%)	3.7 (1.4, 9.9)	3.3 (1.1, 9.6)	**0.028**
	No	123 (37.2%)	208 (62.8%)	1	1	
Polygamous husband	Yes	8 (88.9%)	1 (11.1%)	13.3 (1.6, 107.7)	13.6 (1.1,163)	**0.039**
	No	128 (37.5%)	213 (62.5%)	1	1	
Previous disease	Yes	22 (59.5%)	15 (40.5%)	2.6 (1.3, 5.1)	2.4 (1.1, 5.3)	**0.025**
	No	114 (36.4%)	199 (63.6%)	1	1	

## Discussion

This study identifies the prevalence of antenatal depression and its associated factors, including sociodemographic, obstetric, psychosocial, psychological, and disease and treatment-related factors in rural Bangladesh. The findings reveal a 39% (38.9%, 95% CI = 33.9% to 44%) point prevalence of antenatal depression, with key contributing factors including experiences of sexual violence, unplanned pregnancy, being married to a polygamous husband, pre-existing diseases or health conditions (diabetes mellitus, hypertension, bronchial asthma, and thyroid disorders), and gestational age.

The prevalence observed in this study aligns with findings from other low (34.0%, 95% CI = 33.1% to 34.9%) and middle-income countries (22.7%, 95% CI = 20.1% to 25.2%) [29]. It is similar to the prevalence reported in Pakistan (32.2%, 95% CI = 23.11% to 42.87%) but lower than those in Nepal (50%, 95% CI = 35.64% to 64.36%) and Afghanistan (78.5%) [13, 30]. However, it is higher than that in India (17.74%, 95% CI = 11.19% to 26.96%), and Sri Lanka (12.95%, 95% CI = 8.29% to 19.68%) [30].

The results also correspond with previous research in Bangladesh by Gausia et al. (33%, 95% CI = 27.6% to 37.5%) (14) and Tasnim et al. (36.2% in patients with GDM) [31]. However, it is higher than the prevalence observed in the study by Nasreen et al. (18.3%, 95% CI = 15.9% to 20.7%) (15). A potential explanation for this discrepancy might be differences in the study period, sample size, and location. While this study was conducted in a southern subdistrict of Bangladesh, the study by Nasreen et al. was conducted more than a decade ago in Mymensingh, the north-central region of Bangladesh [15].

Violence remains a significant issue in Bangladesh, where a high proportion of women experience sexual violence [32]. Approximately 37% of urban women and half of rural women experience lifetime sexual violence [33]. Alarmingly, many women believe their husbands have the right to discipline them physically. This study identifies sexual violence as a major contributor to antenatal depression, consistent with research by Peltzer et al. in Thailand [34], Maria Atif et al. in Pakistan [35], and Insan et al. in Bangladesh [16].

Unplanned pregnancy is recognized as a significant factor contributing to antenatal depression among Indian women [36]. Studies by Surkan et al. in northwestern Bangladesh and Gausia et al. in eastern Bangladesh have found a significant association between unwanted pregnancies and antenatal depression, aligning with the findings of this study [14,37].

Among obstetric factors, gestational age is significantly associated with prenatal depression. This study reveals a 60% lower likelihood of depression during the second trimester compared to the first. A systematic review and meta-analysis by Okagbue et al. highlight that the prevalence tends to decrease between the 13^th^ and 28^th^ weeks of gestation [38], similar to our study. These findings reveal valuable information about the participants’ marital and reproductive histories, providing a comprehensive understanding of factors influencing antenatal depression in this population.

While some studies link inadequate family support and male-child preference to antenatal depression [14,16,39], our study finds no such associations. It suggests that, over time, families may have become more attentive and supportive of pregnant mothers. Additionally, the preference for male children appears less pronounced in present-day society. Notably, 82% of women report feeling no pressure to have a male child.

Apart from previous research, this study identifies significant associations between a pre-existing disease and depression during pregnancy. It highlights that having a polygamous husband is also associated with prenatal depression. While Nasreen et al. found a positive correlation between prior depression and prenatal depression [15], our findings show that not only previous mental health disorders but also other pre-existing medical conditions such as diabetes mellitus, hypertension, bronchial asthma, and thyroid disorders contribute to the development of antenatal depression.

With nearly two in five women in rural Bangladesh affected by antenatal depression, these findings underscore the urgent need to raise awareness among healthcare professionals and families to provide enhanced mental health support to pregnant women, particularly during the first and last trimesters of gestation. Targeted interventions should focus on reducing intimate partner violence, discouraging polygamy, and providing additional care for expectant mothers with pre-existing health conditions. Additional counseling for women with unplanned pregnancies is also crucial to improving maternal mental health.

## Limitations

This study has certain limitations, primarily due to a small sample size resulting from time and resource constraints. Participants were recruited from selected government and private hospitals in a remote subdistrict in Bangladesh. However, it is vital to consider that a subset of women might not attend antenatal check-ups unless they experience severe health challenges. Therefore, the findings may not fully capture the diversity of the population, leaving a generalizability bias to the obtained results.

## Conclusion

In conclusion, the prevalence and associated factors of antenatal depression in rural Bangladesh highlight a significant public health concern due to its adverse effects on both maternal and neonatal health. Antenatal depression remains a common issue in rural areas of Bangladesh. To effectively tackle this issue, the government, stakeholders, and policymakers should collaborate on organizing national programs and health education campaigns to raise community awareness. By addressing root causes and developing effective policies and interventions, we can work towards reducing the burden of antenatal depression and ensuring a more positive pregnancy experience for expectant mothers.

## Supporting information

S1 File Characteristics of pregnant women in Lohagara, Narail, 2024. (XLSX)
